# Community health workers: A key to halting Africa’s mpox outbreak

**DOI:** 10.4102/jphia.v16i4.1439

**Published:** 2025-05-28

**Authors:** Ngashi Ngongo, Yap Boum, Kyeng Mercy, Landry D. Tsague, Wazih N. Cho, Gervais L. Folefack Tengomo, Abou Beckr Gaye, Laura N. Ambe, Nebiyu Dereje, Jean Kaseya

**Affiliations:** 1 Africa Centres for Disease Control and Prevention (Africa CDC), Addis Ababa, Ethiopia; 2 World Health Organization (WHO), African Region, Brazzaville, Congo

## Introduction

In August 2024, the Africa Centres for Disease Control and Prevention (Africa CDC) and World Health Organization (WHO) declared mpox a Public Health Emergency of Continental Security (PHECS) and International Concern (PHEIC), respectively.^1^ The declaration elevated mpox to a continental priority, enabling rapid resource mobilisation, political commitment, and coordination among countries and international partners under a unified response framework. In accordance with the Africa CDC statutes granting it authority to declare and coordinate responses to continental public health emergencies,^2^ a unified continental mpox outbreak response was established across Africa and endorsed by African heads of state and government.^3^ The response followed the four ‘One’ principle: One Team co-led by Africa CDC and WHO, collaborating with partner organisations; One plan for implementation through 10 response pillars (coordination, risk communication and community engagement, surveillance, laboratory testing and sequencing, infection prevention and control, vaccination, case management, continuity of services, logistics, and research and innovation); One budget estimated at $599 million to address the needs of countries and provide technical support from partner organisations; and One monitoring and evaluation framework to help countries and partner organisations track progress towards controlling mpox.^4^ Key to the implementation of the plan was the deployment of community health workers (CHWs) in high-burden countries in hotspot areas. The role of CHWs has been critical in controlling both localised and widespread outbreaks, ranging from active case search, contact listing and monitoring, community engagement, community-based infection prevention and control, home-based care and case referrals among others. Such a huge task required both government commitment as well as strong partner coordination efforts at all levels.

## A remarkable response from the international community

Countries developed comprehensive mpox response and vaccination plans in response to the outbreak.^5^ The international community mobilised support from 28 organisations, with many deploying staff to the Incident Management Support Team (IMST) in Kinshasa, Democratic Republic of Congo.^6^ By February 2025, $1.1 billion had been pledged, with $700 million committed or disbursed, mostly towards vaccine procurement, surveillance, laboratory strengthening, health worker training (including CHWs), and community engagement. Vaccine access expanded with 6 million doses secured through Gavi, United Nations International Children’s Emergency Fund (UNICEF), and high-income countries^7,8^; 1.5 million doses allocated to 12 countries through the Access and Allocation Mechanism (AAM) chaired by WHO, 818 600 doses shipped to 8 countries, and 544 000 doses administered. Surveillance was bolstered in 21 countries, with 351 epidemiologists and surveillance officers trained. Laboratory capacity was scaled up significantly: over 175 000 test kits were distributed to 29 countries, and 169 laboratory personnel were trained in diagnostics and biosafety. The number of laboratories capable of diagnosing mpox increased from 8 to 23 in the Democratic Republic of Congo (DRC) and from 1 to 30 in Burundi, demonstrating successful decentralisation of diagnostics.

## Fundamental gaps persist

Despite this progress, a joint intra-action review (IAR) held in Addis Ababa in December 2024 revealed persistent gaps.^5^ By late February 2025, 6 of the 23 affected countries had reached the control phase with no new confirmed cases for 6 weeks, and 8 more showed declining case trends. However, 15 countries still reported active transmission, averaging 2981 suspected and 651 confirmed cases weekly, 8% and 33% rise, respectively, from the previous quarter. DRC and Uganda recorded the highest incidence, with 1.3 and 2.8 confirmed cases per million, respectively. Uganda had the continent’s highest incidence in epidemiological week 10 of 2025.

Challenges remain in surveillance, particularly in the DRC, where only 20% of cases are reported through community systems. Delays of up to 90 days between sample collection and results prolong household and community exposure, prompting reliance on informal care options such as traditional and religious healers. The humanitarian crisis in DRC also increases the risk of cross-border spread, now observed in new countries such as South Sudan and Tanzania.

Compounding this, a freeze in United States (US) government funding has curtailed testing capacity by hampering sample transportation logistics. In response, the WHO International Health Regulations (IHR) Emergency Committee and the Africa Emergency Consultative Group (ECG) extended the PHECS and PHEIC designations, calling for intensified efforts while recognising progress made.

## Back to basics

The IAR underscored a critical weakness: insufficient community engagement and limited deployment of CHWs. To address this, countries such as Burundi, Uganda, and DRC deployed more than 3000 CHWs to bolster surveillance, vaccination, risk communication, and infection prevention. Sierra Leone’s ‘Operation Find Them All’ campaign, launched in February 2025, intensified active case searches in Western Area Urban and Rural districts, leading to a spike in confirmed cases. In the DRC, contact listing surged sevenfold over 3 months, highlighting the effectiveness of CHW-led outreach.

These CHWs, supervised by head nurses and supported by field epidemiologists, conduct house-to-house visits to identify, isolate, and monitor suspected cases. Their efforts have proven vital in disrupting transmission chains and enabling early intervention, particularly in resource-limited and remote settings where traditional systems struggle to reach.

Importantly, CHWs have consistently demonstrated their value in previous public health emergencies.^9^ During the Ebola outbreaks in West and Central Africa, they facilitated community trust, ensured safe burials, and traced contacts effectively. In the COVID-19 pandemic, CHWs played a key role in awareness campaigns, vaccine delivery, and home-based care. In Uganda and Ghana, CHWs supported Marburg virus detection and education. For human immunodeficiency virus (HIV), CHWs have underpinned decades of community-based testing, adherence support, and stigma reduction.

Beyond emergencies, CHWs are central to the theory of change for achieving Universal Health Coverage (UHC). Their proximity to communities, ability to address social determinants of health, and role in health education make them indispensable actors in strengthening primary healthcare systems and reaching underserved populations.^9^

## The way forward

The mpox response has now entered a new phase focused on intensification, integration, and legacy, which prioritises CHWs at the frontlines of surveillance, community engagement, and vaccination campaigns. Integration ensures the coherence of surveillance, laboratory capacity, case management, infection prevention and control, risk communication, and vaccination. In many countries, CHWs serve as a flexible workforce managing multiple conditions simultaneously such as mpox and Ebola in Uganda, or mpox, chickenpox, and measles in DRC and Burundi.

Looking ahead, sustaining CHWs through digital tools and fair remuneration will be essential. Institutionalising CHWs within national epidemic preparedness strategies will secure long-term benefits. Their trusted status in communities, capacity for early detection, and ability to bridge formal health systems and communities make them indispensable to outbreak response.

The response to mpox underscores a broader imperative: community health workers must be embedded in epidemic response planning from the start. Countries need guidance on the minimum standards, including the number of CHWs to deploy during outbreak response and those to sustain as part of preparedness. The current mpox experience can inform outbreak-specific ratios. For instance, the deployment of over 3000 CHWs in three countries covering hotspot zones may offer an emerging benchmark for emergency operations. If such a ratio can be extrapolated, it would contribute to the body of knowledge on health workforce planning in epidemics.

In conclusion, mpox has reaffirmed the indispensable role of CHWs, not just as stopgap responders, but as a key pillar of public health. We must challenge outdated concepts of vertical emergency response and instead invest in the foundations of resilience. Their inclusion at the outset will accelerate containment and mitigate the human, social and economic toll of emerging epidemics. Africa’s response to mpox offers an opportunity to transform CHWs into the cornerstone of public health resilience across the continent.
